# Reverse Gradient Distributions of Drug and Polymer Molecules Within Electrospun Core–Shell Nanofibers for Sustained Release

**DOI:** 10.3390/ijms25179524

**Published:** 2024-09-01

**Authors:** Yaoning Chen, Wenjian Gong, Zhiyuan Zhang, Jianfeng Zhou, Deng-Guang Yu, Tao Yi

**Affiliations:** 1 School of Materials and Chemistry, University of Shanghai for Science and Technology, Shanghai 200093, China; 222203082@st.usst.edu.cn (Y.C.); 223353279@st.usst.edu.cn (W.G.); 223353170@st.usst.edu.cn (Z.Z.); 221550217@st.usst.edu.cn (J.Z.); 2 Faculty of Health Sciences and Sports, Macao Polytechnic University, Macau 999078, China

**Keywords:** molecular gradient distribution, coaxial electrospinning, core–shell nanofibers, sustained release, poorly water-soluble drugs

## Abstract

Core–shell nanostructures are powerful platforms for the development of novel nanoscale drug delivery systems with sustained drug release profiles. Coaxial electrospinning is facile and convenient for creating medicated core–shell nanostructures with elaborate designs with which the sustained-release behaviors of drug molecules can be intentionally adjusted. With resveratrol (RES) as a model for a poorly water-soluble drug and cellulose acetate (CA) and PVP as polymeric carriers, a brand-new electrospun core–shell nanostructure was fabricated in this study. The guest RES and the host CA molecules were designed to have a reverse gradient distribution within the core–shell nanostructures. Scanning electron microscope and transmission electron microscope evaluations verified that these nanofibers had linear morphologies, without beads or spindles, and an obvious core–shell double-chamber structure. The X-ray diffraction patterns and Fourier transform infrared spectroscopic results indicated that the involved components were highly compatible and presented in an amorphous molecular distribution state. In vitro dissolution tests verified that the new core–shell structures were able to prevent the initial burst release, extend the continuous-release time period, and reduce the negative tailing-off release effect, thus ensuring a better sustained-release profile than the traditional blended drug-loaded nanofibers. The mechanism underlying the influence of the new core–shell structure with an RES/CA reverse gradient distribution on the behaviors of RES release is proposed. Based on this proof-of-concept demonstration, a series of advanced functional nanomaterials can be similarly developed based on the gradient distributions of functional molecules within electrospun multi-chamber nanostructures.

## 1. Introduction

One of the most important aspects of the nanotechnology era is that molecules can be organized to create nanostructural products to achieve desired outcomes [1,2,3,4]. Based on the organization routes, nanotechnologies are categorized into three kinds: bottom-up methods from molecules to nanoscale products (such as molecular self-assembly or self-aggregation) [5], top-down methods from macroscale materials to nanoscale products (such as electrospinning and electrospraying [6,7,8,9]), and their combinations (such as molecular self-assembly with electrospun nanofibers used as templates to manipulate the aggregation of molecular building blocks within a nanoscale confinement region). By tailoring their accurate spatial positioning within nanoproducts, the molecules of an active ingredient can be enhanced to provide better or even new functional applications [10,11].

The sustained release of active ingredients for longer functional time periods is a hot topic in a wide variety of applied scientific fields, such as drug delivery, tissue engineering, cosmetics, agriculture, and public health [12,13,14,15,16]. Regarding the sustained release of drug molecules in pharmaceutics, the most common approach is to encapsulate the drug molecules in insoluble or biodegradable polymeric matrices or lipids, resulting in the successful development of some commercial nanoproducts for human well-being [17,18,19,20,21,22,23,24,25,26]. New excipients, methods, techniques, and strategies are continuously sought to provide enhanced sustained-release effects, such as the entrapment of drug molecules in inorganic carriers (e.g., graphene, SiO_2_, or MXene), drug–polymer conjugates, drug-release-initiating nanogates, loading into new supramolecular substances (e.g., MOF, COF, or ZIF), and sealing within polymeric nanostructures such as dendrimers [27,28,29,30,31].

Among all the types of complicated polymer-based nanostructures, the core–shell structure is the most fundamental one [32,33,34]. Its simple inner–outer spatial relationship is able to greatly enrich the strategies for conceiving advanced functional materials with a wide variety of functional applications [35,36,37], including the sustained release of drug molecules. In the literature, there are abundant investigations into the sustained release of drugs from core–shell nanoparticles [38,39] and there are many review articles focusing on this topic [40,41,42,43]. Following this trend, several studies have examined electrospun core–shell nanofibers with the aim of expanding their potential applications in improving the sustained-release behaviors of certain drug molecules [44,45,46].

During its development history, sustained drug release from electrospun nanofibers has gone through several key stages: (1) insoluble or biodegradable polymeric nanofibers or cross-linked water-soluble polymers as matrices [47,48,49,50,51]; (2) core–shell nanostructures from coaxial electrospinning processes, in which the shell sections from electrospinnable fluids were explored as a barrier to extend the release of drug molecules [52]; (3) hydrophobic or insoluble polymer coatings on drug–polymer nanocomposites, which were realized through so-called modified coaxial electrospinning (e.g., unspinnable CA and lipids were reported to ensure better sustained release as a shell coating layer) [53,54]; (4) elaborate designs of the core components and compositions, such as drug nanodepots for sustained drug release; and (5) tri-layer core–shell structures and other complicated products created via a combined strategy, which can be viewed as a derivative of the traditional double-chamber core–shell structure [55,56,57,58].

There are no limitations for nanostructures, as a main driving force of nanoscience and nanotechnology, to provide strategies for the development of novel sustained-drug-release materials. In the present study, we hypothesized the possible formation of two gradient distributions of the host polymeric molecules and guest drug molecules within electrospun core–shell nanofibers. With cellulose acetate (CA) as the key polymeric matrix and resveratrol (RES) as a model active ingredient, a decreasing gradient distribution of CA and an increasing gradient distribution of RES from the periphery to the center were introduced within electrospun core–shell nanofibers. These reverse radial gradient distributions of the drug and polymer are expected to provide a better sustained-release profile, which can be evaluated on the basis of the initial burst effect, the sustained-release time period, and the negative leveling-off effect. The drug release mechanism and the related process–structure–property relationship were also determined.

Our reasons for selecting CA as the key polymeric matrix are that it is a derivative polymer from natural cellulose that has excellent properties for sustained drug release due to its insolubility, bio-compatibility, and easy processing capability [59,60,61,62]. RES, as a naturally occurring non-flavonoid molecule, is a well-known model drug in the literature. It has been reported to have the potential to cure a wide variety of diseases, including cardiac, pulmonary, vascular, and neurodegenerative diseases, as well as diabetes, cancer, obesity, and arthritis. However, RES has low water solubility (about 0.03 mg/mL) and low oral bioavailability (considerably less than 1%) due to its short biological half-life [63]. Sustained release is desired for RES to play its role in treating the abovementioned diseases [64,65,66].

## 2. Results and Discussion

### 2.1. Key Elements for Implementing Coaxial Electrospinning

**Figure 1:**
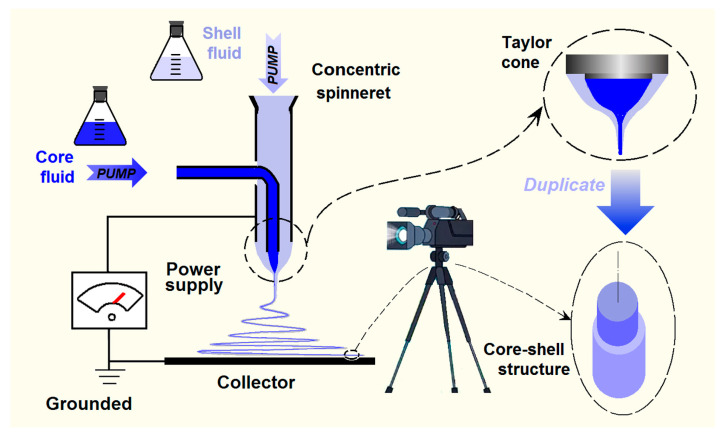
A diagram of coaxial electrospinning and its implementation.

Coaxial electrospinning was once regarded as one of the most important breakthroughs in the field of electrohydrodynamic atomization (EHDA) [67]. This is because it caters to the developments of nanoscience and nanotechnology, in which core–shell nanostructures represent one of the most popular topics in the development of novel functional nanomaterials [68,69,70]. As shown in Figure 1, a coaxial electrospinning system has four main parts: two pumps, a power supply, a spinneret, and a collector. Single-fluid blending electrospinning can also be performed, provided that either the core or shell fluid is switched off. However, compared with traditional blending electrospinning, coaxial electrospinning requires special key elements for successful operation. First of all, the two fluids must be sufficiently compatible to be co-jetted without coagulation or even reaction. Secondly, one of the two fluids must be electrospinnable to dominate the whole process of creating an integrated core–shell structure and maintaining a linear morphology. Thirdly, a concentric spinneret with a reasonable design is very important for implementing these processes and for the formation of core–shell nanostructures. This is because the concentric nozzle of the spinneret is the macro template for the core–shell nanostructure; it also exerts a remarkable influence on the formation of the compound Taylor cone, by which the coaxial electrospinning working process is initiated.

**Figure 2:**
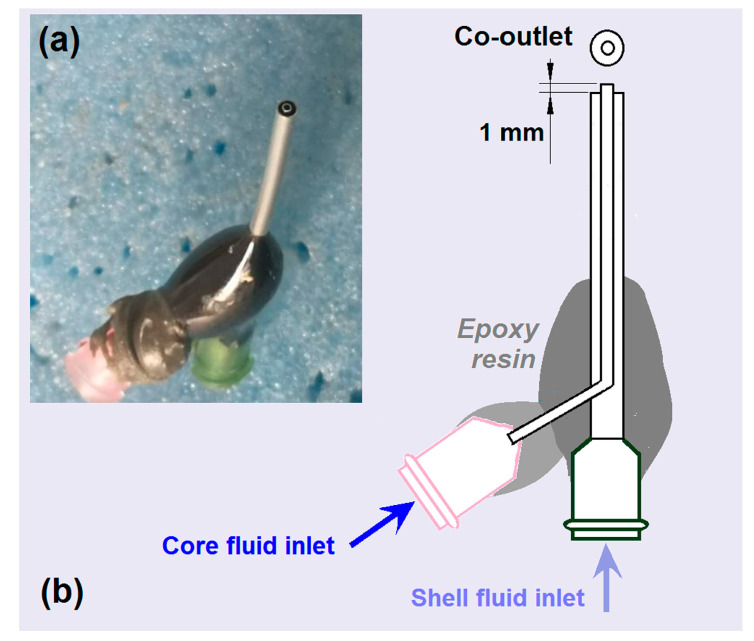
The homemade concentric spinneret: (**a**) a digital image and (**b**) a diagram showing the inner structure and connections.

Among the four parts of the electrospinning system, the spinneret is the most important section as it is associated with the greatest innovations [71,72,73]. In this investigation, a homemade spinneret was prepared for use in all the electrospinning processes. The spinneret had a low weight of 2.12 g. A digital image of the spinneret and a diagram of its inner structure are included in Figure 2. As indicated in Figure 2a, most of the surface of the spinneret was covered by a polymer to provide insulation and prevent energy loss to the environment [74]. The inner stainless steel capillary was arranged to project about 1 mm beyond the outer metal capillary at the co-outlet nozzle (Figure 2b). This design is conducive to the encapsulation of core fluid by shell fluid, and it also inhibits the diffusion of molecules between the core and shell working fluids after they are pumped out and are under the effect of the electrical field.

**Figure 3:**
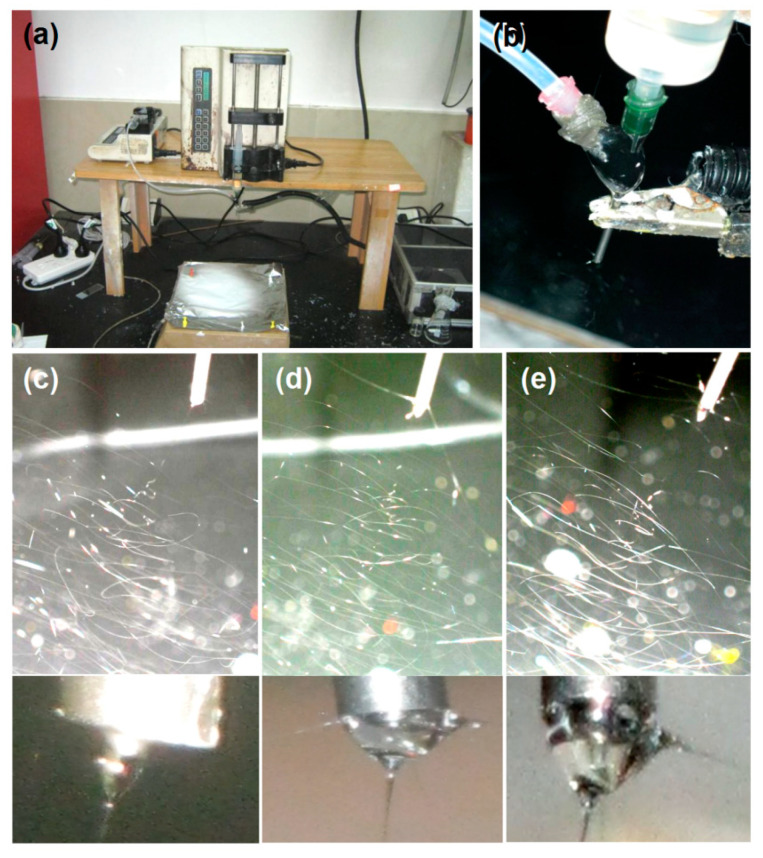
The homemade electrospinning apparatus and observations of different working processes: (**a**) an image of the entire electrospinning system; (**b**) the convergence point of the two working fluids and the transferal of electrostatic energy; (**c**–**e**) typical working processes for fabricating nanofibers F2, F1, and F3, respectively. The insets at the bottom show the typical Taylor cones.

A digital image of the entire homemade electrospinning apparatus is given in Figure 3a. Two syringe pumps (one a KDS100 and the other a KDS200) were employed to quantitatively drive the core and shell working fluids to the concentric spinneret. The collector was simply prepared by placing aluminum foil on hard cardboard and was grounded through an alligator clip. This is useful for safe operation and also for instantly removing the electrostatic energy on the collected solid nanofibers, which, in turn, is useful for the deposition of later-formed solid nanofibers. The spinneret was the convergence point of the two working fluids and the transferal of electrostatic energy through the alligator clip (Figure 3b). It was also the starting point of the working process, characterized by the formation of the Taylor cone, which always hung below the nozzle of the spinneret.

Both CA and polyvinylpyrrolidone (PVP) are inert polymers that are popular in pharmaceutics and have excellent filament-forming properties [75,76]. Based on their properties and some pre-optimization experiments, the two working fluids, i.e., Fluid 1 (shell fluid, composed of 10% (*w*/*v*) CA, 5% (*w*/*v*) PVP, and 5% (*w*/*v*) RES in a solvent mixture of acetone, ethanol, and N, N-dimethylacetamide (DMAc) with a volume ratio of 4:1:1) and Fluid 2 (core fluid, consisting of 5% (*w*/*v*) CA, 5% (*w*/*v*) PVP, and 10% (*w*/*v*) RES in the above-mentioned solvent mixture), were determined. Three kinds of medicated nanofibers were prepared: homogeneous nanofibers of the shell fluid (F1), homogeneous nanofibers of the core fluid (F2), and heterogeneous core–shell nanofibers (F3) from the co-treatment of the shell and core fluids in a coaxial manner. The applied voltage, fluid flow rate, and fiber collection distance are included in Table 1. The ambient temperature and humidity were 24 ± 4 °C and 51 ± 7%, respectively.

The typical working processes for the preparation of F2 nanofibers from the core capillary and F1 nanofibers from the shell capillary are exhibited in Figure 3c and Figure 3d, respectively. Their Taylor cones were clearly visible, as shown in the bottom insets. After a straight fluid jet (which was too small to discern), the initiation and gradual enlargements of the bending and whipping loops to draw the fluid jets into solid nanofibers in the unstable region were clearly visible. Similarly, when both the core and shell fluids were pumped out of the spinneret nozzle, the coaxial working procedure was clear, as shown in Figure 3e. Although both fluids were almost transparent under the strong magnesium light of the camera, their different transmittances ensured the visibility of the core–shell Taylor cone (the bottom inset of Figure 3e). The red and yellow dots in these images resulted from the thumbtacks that were utilized to fix the aluminum foil onto the hard cardboard (Figure 3a).

### 2.2. The Electrospun Nanofibers’ Morphology and Inner Structure

**Figure 4:**
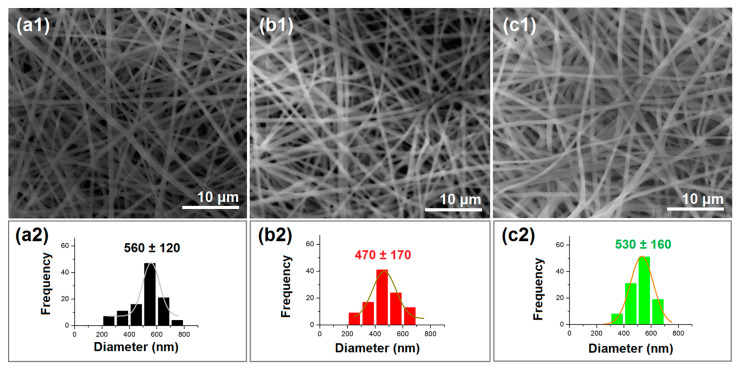
SEM images of the prepared nanofibers and their size distributions: (**a1**,**b1**,**c1**) are images of nanofibers F1, F2, and F3, respectively; (**a2**,**b2**,**c2**) are their corresponding diameter distributions.

Scanning electron microscope (SEM) images of the prepared homogeneous nanofibers (F1 and F2) and core–shell nanofibers (F3) are given in Figure 4a1, Figure 4b1 and Figure 4c1, respectively. It is clear that all these nanofibers had typical linear morphologies. No beads or spindles could be discerned on them, suggesting robust and stable electrospinning processes. Based on measurements made using ImageJ software V1.8.0, the F1, F2, and F3 nanofibers had average diameters of 560 ± 120 nm, 470 ± 170 nm, and 530 ± 160 nm, as shown in Figure 4a2, Figure 4b2 and Figure 4c2, respectively. The F1 nanofibers had a significantly larger diameter than the F2 nanofibers. This can be attributed to the generally higher combined concentration of the two polymers for F1 (i.e., a total of 15% CA and PVP) than for F2 (10% CA and PVP). Dual influences of the drug concentration on the fibers’ diameters are anticipated. On the one hand, the nanofibers’ diameter might increase due to the increase in the amount of solutes in the working fluids. On the other hand, the drug, as a small molecule, might increase the conductivity of the working fluid and, in turn, strengthen the electrical drawing processes to reduce the nanofibers’ diameter. These effects will be systematically investigated in a future study. As expected, the F3 core–shell nanofibers had an average diameter value that was between those for the F1 and F2 monolithic nanofibers.

**Figure 5:**
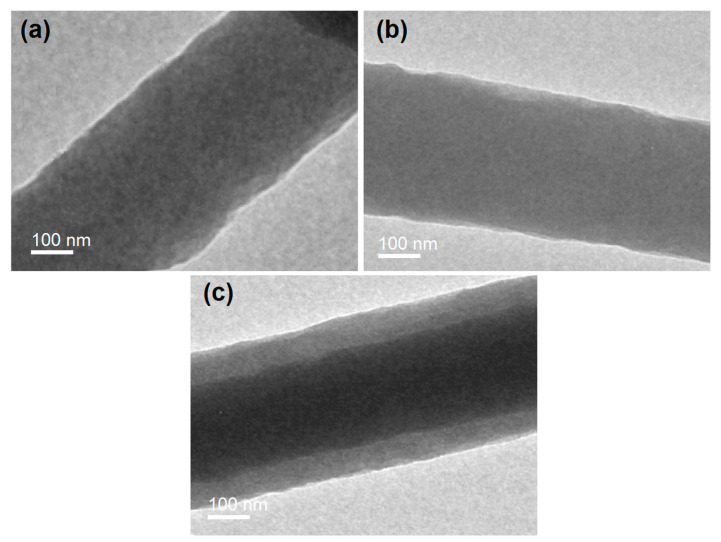
TEM images of the prepared nanofibers: (**a**) homogeneous F1, (**b**) homogeneous F2, and (**c**) core–shell F3 nanofibers.

TEM images of the F1, F2, and F3 nanofibers are exhibited in Figure 5a, Figure 5b and Figure 5c, respectively. As indicated by the uniform gray levels in the images in Figure 5a,b, the F1 and F2 nanofibers from the single-fluid blending processes were homogeneous nanocomposites, with drug molecules distributed evenly within the polymeric matrices. Most probably, they presented in the nanofibers in a molecular manner, propagated from the corresponding solutions. The homogeneous state in the solution was successfully maintained in the solid nanofibers due to the extremely fast drying processes involved in electrospinning [77,78,79].

In sharp contrast, the F3 core–shell nanofibers had obviously differing gray levels for the core and shell sections (Figure 5c). This is likely due to the filling effects of the high-concentration RES molecules in the core sections; additionally, the core section had a larger thickness, which resulted in a significantly higher gray level compared to that for the shell section. Based on Figure 5c, the core section had an estimated diameter of 250 nm, and the shell section had an average thickness of 80 nm. Given the same general solute concentrations of the core and shell fluids, i.e., a total of 20% (*w*/*v*) CA, PVP, and RES, and the same flow rate of 1.0 mL/h, the density ratio of the core section to the shell section should be inversely proportional to their surface area ratio. The value can then be calculated as {[(125 + 80)^2^π − (125^2^π)]/(125^2^π)} × 100% = 168.96%. This higher density value of the core section suggests that the small RES molecules filled the voids between the polymeric molecules and significantly elevated the packing density of the core nanocomposites.

### 2.3. The Physical State of the Components and Their Compatibility

**Figure 6:**
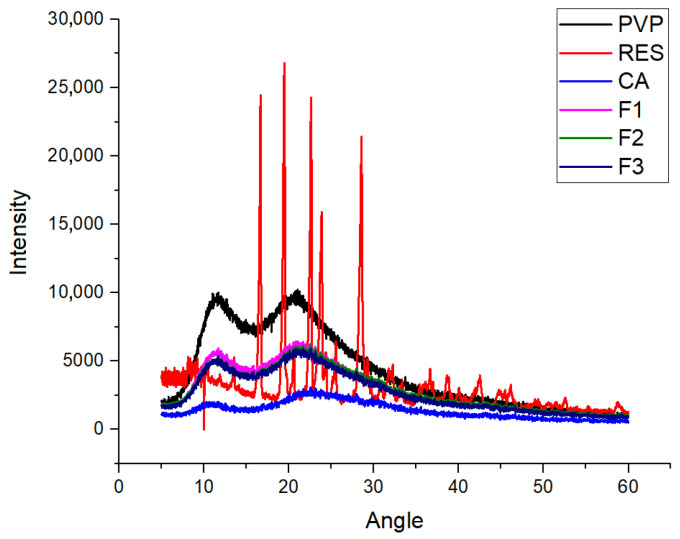
XRD patterns of the three raw components (RES, PVP, and CA) and the different kinds of electrospun nanofibers (F1, F2, and F3).

The two fundamental states of solid materials are crystalline and amorphous. For the effective dissolution and delivery of a poorly water-soluble drug, its amorphous state is advantageous, particularly its distribution on a molecular scale within a polymeric matrix. In pharmaceutics, such drug–polymer composites are termed amorphous solid dispersions or molecular solid dispersions [80,81]. X-ray diffraction (XRD) is the most frequent method used to disclose the physical state of components within a nanoscale product [82,83,84]. The patterns of the raw materials of CA, PVP, and RES and their fibrous products F1 to F3 are exhibited in Figure 6. As anticipated, the drug RES exhibited a series of sharp Bragg peaks in its diffraction patterns, indicating that it presents in a crystal state initially. Both PVP and CA are amorphous polymeric matrices, as shown by their blunt halos. When they were converted into monolithic composite nanofibers (F1 and F2) or core–shell hybrid nanofibers (F3), they all inevitably presented in an amorphous state. Electrospinning is essentially a physical drying process, and the time cost for the working fluids to be converted into solid nanofibers is within one minute. In the working fluids, the solutes, including the host polymeric matrices and guest drug, are uniform throughout. This homogeneous state in the co-dissolved solutions is propagated into the solid nanofibers via the electrospinning process; thus, amorphous RES can be ensured, whether through single-fluid blending electrospinning or through double-fluid coaxial electrospinning.

**Figure 7:**
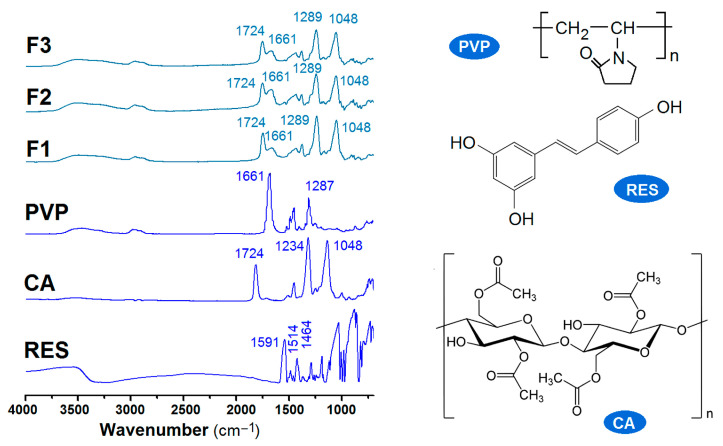
ATR-FTIR spectra of the three raw components (RES, VPV, and CA) and the different kinds of electrospun nanofibers (F1, F2, and F3), along with their molecular formulas.

The amorphous state is a higher energy state that is prone to transfer to a lower thermodynamic level, i.e., a crystalline state, particularly at a high drug content. Favorable compatibility between the guest drug molecule and the host polymeric matrix is crucial to the stability of polymer–drug nanocomposites [85,86,87]. Here, attenuated total reflectance–Fourier transform infrared spectroscopy (ATR-FTIR) was utilized to evaluate the compatibility between RES and CA or PVP. The spectra of the raw materials (CA, PVP, and RES) and the three types of electrospun nanofibers (F1 to F3) are included in Figure 7. According to their molecular formulas, RES has three OH groups per molecule, PVP has many C=O groups within each molecule, and CA has both OH and C=O groups in each molecule. These groups suggest that hydrogen bonds can form between RES and PVP, between RES and CA, and between PVP and CA, indicating that these components are highly compatible. The spectra of the three nanofibers obviously demonstrated these interactions, showing an overlap of PVP and CA. As indicated by its molecular formula, the chemical structure of RES consists of two aromatic rings connected by a methylene bridge and contains three phenolic groups. The benzene rings easily form a large π electron cloud with the long carbon chains within CA and PVP, causing the characteristic peaks to disappear in the spectra of these nanofibers.

### 2.4. In Vitro Dissolution Tests

The drug loading efficiencies (LEs) of the F1, F2, and F3 nanofibers were 98.94 ± 2.09%, 93.66 ± 4.57%, and 97.72 ± 3.17%, respectively. One advantage of electrospun medicated nanofibers for drug delivery applications is their high LE value. During the extremely fast drying process, the drug molecules in the working fluids have very limited chances to escape solidifying into nanofibers together with the polymeric matrix [11,45,53]. It is likely that the only route for drug loss is the evaporation of solvent molecules, by which some drug molecules may simultaneously “evaporate” to the environment. Thus, it can be anticipated that a higher drug concentration in the working fluid (such as for the F2 nanofibers) means a greater possibility of drug loss from the nanofibers and, in turn, a relatively smaller LE value. The coaxial electrospinning process for preparing the F3 core–shell nanofibers, in which a solution with a lower drug concentration was used to encapsulate a solution with a larger drug concentration, was able to effectively prevent drug loss to a certain extent.

**Figure 8:**
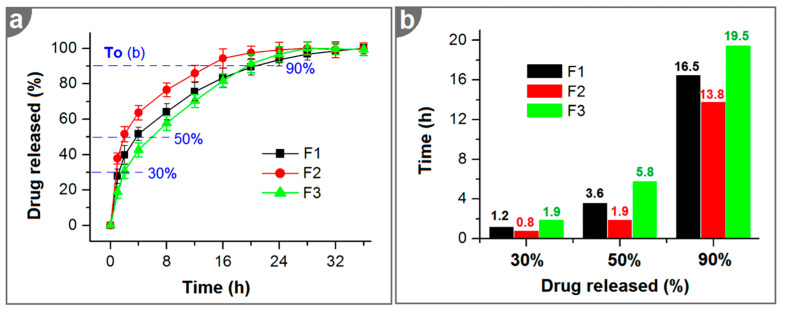
In vitro drug dissolution tests: (**a**) accumulative release profiles of RES from the three kinds of medicated nanofibers, F1, F2, and F3; (**b**) the times needed to release certain amounts of the loaded drug, derived via the interpolation method.

As anticipated, the three types of electrospun nanofibers, whether they were homogeneous composites (F1 and F2) or heterogeneous core–shell structures (F3), were all able to provide a typical RES sustained-release profile, as shown in Figure 8a. Although these in vitro drug release profiles are apparently similar, they are significantly different. Thus, the interpolation method was exploited to determine the times that were needed to release certain percentages of the loaded RES. The percentages 30%, 50%, and 90% are commonly used to evaluate sustained drug release and, in particular, to assess the initial burst release, the extended-release time period, and the severity of tailing-off release. The results are presented in Figure 8b. Compared with the homogeneous nanofibers (F1 and F2), the core–shell nanofibers (F3) provided the best sustained-RES-release profiles. This conclusion was made by considering the following aspects: (i) it took the longest time to reach 30% release, i.e., 1.9 > 1.2 > 0.8, meaning a weaker initial burst release effect; (ii) it took the longest time to reach 50% release, i.e., 5.8 > 3.6 > 1.9, meaning that it had the best effect in extending the RES continuous-release time period; and (iii) it took the longest time to reach 90% release of the loaded RES, i.e., 19.5 > 16.5 > 13.8, suggesting that it has the longest sustained-release time period and the weakest tailing-off release.

### 2.5. Mechanisms of the Reserve Gradient Distributions in Sustained Release

**Figure 9:**
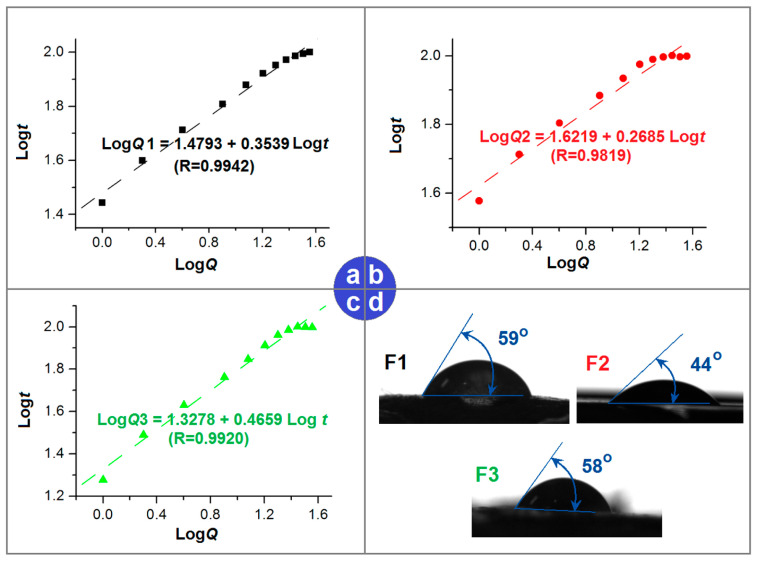
(**a**–**c**) Regressed RES release mechanisms based on the Peppas equation for the F1, F2, and F3 nanofibers, respectively. (**d**) The WCAs for the three kinds of electrospun nanofibers.

The well-known Peppas equation was applied to regress the in vitro dissolution data to determine the kinetic mechanism by which drug molecules were transferred from the nanofibers into the dissolution medium [88]. The equations for F1, F2, and F3 were Log *Q*1 = 1.4793 + 0.3539 Log *t* (R = 0.9942) (Figure 9a), Log *Q*2 = 1.6219 + 0.2685 Log *t* (R = 0.9819) (Figure 9b), and Log *Q*3 = 1.3278 + 0.4659 Log *t* (R = 0.9920) (Figure 9c), respectively. As expected, both the F1 composite nanofibers with a lower drug content and the F2 composite nanofibers with a higher drug content had exponent values smaller than the critical value of 0.45, i.e., 0.3539 < 0.45 and 0.2685 < 0.45. Thus, RES release from the F1 and F2 electrospun blended nanofibers was governed by a typical Fickian mechanism. However, the F3 electrospun core–shell nanofibers had a value of 0.4659, slightly larger than 0.45, hinting that their RES release was governed by a combination of diffusion and erosion mechanisms. This abnormal phenomenon is likely related to the double-chamber structures and the gradient distributions. One of the prerequisites of the Peppas equation is the homogeneous distribution of drug molecules within the polymeric matrix, which holds in the cases of F1 and F2 but not that of F3.

The surface properties of the nanofibers also play a role in the behavior of RES release; these properties are closely related to the components and compositions of the products. As shown in Figure 9d, the F1, F2, and F3 electrospun nanofibers had water contact angles (WCAs) of 59, 44, and 58 degrees, indicating that these nanofibers had fine hydrophilicity. CA is a hydrophobic polymer, and RES is insoluble; thus, it is the presence of PVP that improves the hydrophilicity of these nanofibers. In the literature, electrospun CA nanofibrous mats showed hydrophobicity with a WCA of 91.7° [62]. The addition of PVP had several positive effects in this study, besides its excellent filament-forming properties. One of these was improving the hydrophilicity; the second was forming pores for the penetration of water molecules and the diffusion of RES molecules; and the third was improving the solubility of RES, resulting in higher and more stable drug concentration differences between the swollen nanofibers and the dissolution bulk medium. In the literature, PVP is reported to be able to improve the saturated solubility of over 200 poorly water-soluble drugs [89]. Additionally, compared with the initial weights of nanofibers placed into the dissolution vessels, the weights of residual nanofibers after the in vitro dissolution processes had finished were, on average, 57.46 ± 6.54%, 27.14 ± 3.47%, and 48.29 ± 4.66% for F1, F2, and F3, respectively. The RES was exhausted. The CA contents in the solid nanofibers were 50%, 25%, and 37.5% for F1, F2, and F3, respectively. Thus, the proportions of PVP dissolved into the dissolution medium were 70.16%, 91.77%, and 56.84%, respectively. This result suggests that the compact shell section also prevented the dissolution of PVP molecules from the cores of the nanofibers into the dissolution medium.

**Figure 10:**
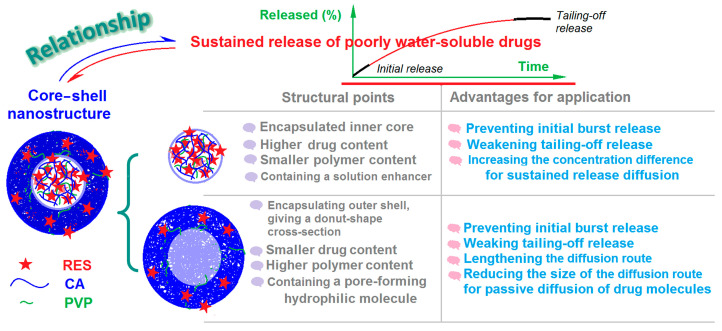
A diagram of the molecular distributions within the core–shell nanostructures and their advantages for ensuring an improved sustained drug release profile.

In the literature, there have been numerous investigations into the process–structure–performance relationship for core–shell nanostructures, including both nanofibers and nanoparticles. Here, the core–shell structure with reverse gradient distributions of drug and polymer molecules can be viewed as a derivative nanostructure of the common core–shell nanostructure. Correspondingly, this new nanostructure possesses a series of structural characteristics and related advantages that ensure the desired functional performance. These contents are shown in Figure 10, which also presents a molecular mechanism for the drug molecules that links reasonable distributions to the desired release behaviors.

A higher drug content and a lower CA content in the core section are useful for preventing the initial burst release. Meanwhile, the co-presence of PVP, as a RES dissolution enhancer, is conducive to an increased drug concentration difference for stable and continuous diffusion from the fibers to the bulk dissolution medium; it is also a positive factor in reducing the tail-off in release. For the shell section, the lower drug concentration means a low initial release, which is similarly useful for preventing the initial burst release effect. Meanwhile, the higher concentration of CA means a longer route and a smaller pore size for the penetration of water molecules and the passive diffusion of RES molecules, which is conducive to a more stable, more continuous, and longer-lasting sustained-release effect. The presence of PVP molecules in the shell section can also act to promote the formation of pores through which the drug molecules can diffuse outward.

## 3. Materials and Methods

### 3.1. Materials

Cellulose acetate (CA, *M*_w_ = 50,000), polyvinylpyrrolidone (PVP) K60 (*M*_w_ = 130,000), anhydrous ethanol, acetone, and N, N-dimethylacetamide (DMAc) were purchased from Sigma-Aldrich (Shanghai, China). Resveratrol (purity: 98%) was purchased from Shanghai Haosheng Biomed. Co., Ltd. (Shanghai, China). Phosphate buffer solution (PBS, 0.1 M, pH = 7.0) was supplied by Tianjin Zhiyuan Chemical Reagent Co., Ltd. (Tianjin, China). All chemicals and reagents were used without any additional treatment, and water was double-distilled just before use.

### 3.2. Electrospinning

A homemade electrospinning apparatus, characterized by a homemade concentric spinneret, was employed for all the preparations. The other components of the apparatus included two syringe pumps (one KDS 100 and one KDS200, Cole-Parmer, Holliston, MA, USA), a ZGF60 kV/2 mA high-voltage generator (Wuhan Hua-Tian High Power Co, Ltd., Wuhan, China), and a simple collector prepared from aluminum foil and hard cardboard.

### 3.3. Morphology and Inner Structures

A scanning electron microscope (SEM, FEI Quanta 450 FEG, FEI Corporation, Hillsboro, OR, USA) was utilized to observe the external morphologies of all the nanofibers. All the samples were Au-coated for 1 min before they were placed into the SEM chamber, and statistical results regarding the diameter distributions were obtained using ImageJ software V1.8.0 (NIH, Bethesda, MD, USA) and Origin2021. 

A transmission electron microscope (TEM, Thermo Talos F200X G2, Waltham, MA, USA) was used to assess the inner structures of the resultant nanofibers. The samples were prepared by placing a copper-supported carbon film under the spinneret but just above the collector to collect samples for about 10 s.

### 3.4. Physical States and Compatibility

The components’ physical states were assessed using a Bruker D8 Advance X-ray diffractometer (XRD, D8 ADVANCE, Bruker, Karlsruhu, Germany) with a copper target tube under a voltage of 40 kV, a tube current of 40 mA, a scanning rate of 8°/min, and a minimum step size of 0.02°. Attenuated total reflectance–Fourier transform infrared spectroscopy (ATR-FTIR) was performed using a SPECTRUM 100 spectrometer (Perkin Elmer, Billerica, MA, USA). The instrument had a resolution of 1 cm^−1^, the scanning range was 450–4000 cm^−1^, and each sample was scanned 8 times.

### 3.5. Hydrophilicity

The hydrophilicity of the electrospun functional nanofibrous membrane surface was measured using a WCA meter (JY-82B Kruss DSA, Kruss Company, Kruss, Germany). The measurement range was 0–180°, with an angle measurement error of ±0.5°.

### 3.6. In Vitro Dissolution Tests

The drug loading efficiencies (LEs) of the nanofibers were measured according to the following Equation (1):

(1) $$LE\%=\frac{C_{M}}{C_{T}}\times 100\%$$

in which *C_M_* and *C_T_* represent the measured RES concentration in the solid nanofibers and the theoretically calculated RES concentration according to the preparation conditions. *C_M_* values were obtained by dissolving 10.0 mg of fibers into 10.0 mL of 50% ethanol aqueous solution. Subsequently, 1.0 mL of the solution was diluted 20 times with PBS and observed using a UV–vis spectrophotometer (Unico2000, Unico Co., Ltd., Shanghai, China). *C_T_* was 20%, as indicated in Table 1.

In vitro dissolution tests of the blended nanofibers (F1 and F2) and core–shell nanofibers (F3) were carried out to assess the sustained drug release profiles. These tests were performed according to the paddle method described in the Chinese Pharmacopoeia (2020 Ed.). A quantity of nanofibers containing 20 mg RES was immersed in the vessel of a dissolution apparatus (RCZ-8A, Radio Factory, Tianjin University, Tianjin, China) containing 900 mL of PBS at 37 °C and was agitated at a rotation speed of 50 rpm. At predetermined intervals, 5.0 mL aliquots of the release medium were collected from the dissolution vessel, and 5.0 mL of fresh PBS was added to the vessel to maintain a constant volume. The absorbance of RES was measured using a UV–vis spectrophotometer (Unico2000, Unico Co., Ltd., Shanghai, China) at a wavelength of 295 nm. A predetermined standard equation relating the absorbance (A) and drug concentration (C, μg/mL) was used to calculate the concentration of RES: A = 0.3473 × C − 0.03751 (R = 0.9991, range: 1 to 20 μg/mL). The cumulative release percentage was further calculated using the following Equation (2):

(2) $$P(\%)=\frac{C_{n}*V_{0}+\sum_{i=1}^{n-1}C_{i}*V}{Q_{0}}*100$$

where *V*_0_ is the volume of the dissolution medium (900 mL), *V* is the volume of sample withdrawn (5.0 mL), *Q*_0_ is the theoretical amount of the drug in each sample (mg), *C_n_* is the concentration of the drug measured in the nth aliquot (mg/L), and *C_i_* is the concentration of the drug in the *i*-th aliquot (mg/L). After the dissolution tests, the dissolution media were filtered, and the residues were dried in an oven to a constant weight at a temperature of 40 °C.

### 3.7. Statistical Analysis

Unless otherwise specified, all experimental tests were performed in triplicate. Data are expressed as the means ± standard deviations (SDs). A statistical analysis of the data was performed using analysis of variance (ANOVA). The ANOVA was followed by the Dunnett test when appropriate, and a value of *p* < 0.05 was considered to indicate a statistically significant difference.

## 4. Conclusions

A brand-new core–shell nanostructure, characterized by reverse distributions of its constituent drug (RES) and polymer (CA) molecules, was successfully fabricated through a single-step coaxial electrospinning process. Based on the increasing gradient distribution of RES and the decreasing gradient distribution of CA from the shell to the core, the behaviors of RES molecule release were intricately adjusted to prevent the initial burst release effect, extend the continuous-release time period, and reduce the negative tailoring-off release period. Thus, the new core–shell nanostructures provided a better sustained RES release profile than composite drug-loaded nanofibers made via blending electrospinning. In vitro dissolution tests indicated that the core–shell nanofibers were able to extend the time taken to reach 90% release of the loaded RES to 19.5 h, with only 18.9 ± 3.6% release occurring in the first hour. The core–shell structure, the linear morphology, the amorphous state of the components, and their fine compatibility were verified by means of TEM, SEM, XRD, and FTIR measurements. The molecular distribution and the diffusion mechanism to explain the sustained release from the amorphous core–shell nanostructures were proposed. Electrospinning has been successfully demonstrated to be useful for treating a series of polymers and for a wide variety of applications in drug delivery by way of single-fluid blending electrospinning and the resultant monoaxial composites or hybrids [90,91,92,93,94]. Multi-chamber nanostructures currently represent a cutting-edge area of nanoscience and nanotechnology, and they are highly popular in the development of novel functional nanomaterials [95,96,97]. Electrospinning can be used to create many novel multi-chamber nanostructures in a simple and straightforward manner [98,99,100,101]. Designing gradient distributions or other types of distributions (such as discrete or interval ones) of key components within multi-chamber nanostructures can further enrich the available strategies for developing advanced structures and functional nanomaterials.

## Figures and Tables

**Table 1:** Experimental parameters for the preparation of the three kinds of medicated nanofibers.

Sample No.	Electro- Spinning	Operational Parameters ^a^				Drug Content (%)	CA Content (%)
		V (kV)	F (mL/h)		L (cm)		
			Core	Shell			
F1	Blending	11	0.0	2.0	20	25%	50%
F2	Blending	11	2.0	0.0	20	50%	25%
F3	Coaxial	11	1.0	1.0	20	37.5% ^b^	37.5% ^b^

^a^ Fluid 1 (or the shell fluid) was composed of 10% (*w*/*v*) CA, 5% (*w*/*v*) PVP, and 5% (*w*/*v*) RES, while Fluid 2 (or core fluid) consisted of 5% (*w*/*v*) CA, 5% (*w*/*v*) PVP, and 10% (*w*/*v*) RES; a solvent mixture consisting of acetone, ethanol, and DMAc with a volume ratio of 4:1:1 was utilized for preparing all the working fluids. ^b^ This is the general apparent content value of the whole core–shell nanofibers.

## Data Availability

The data supporting the findings of this manuscript are available from the corresponding authors upon reasonable request.
