# Correction: Chen et al.  Reverse Gradient Distributions of Drug and Polymer Molecules Within Electrospun Core–Shell Nanofibers for Sustained Release. *Int. J. Mol. Sci.* 2024, *25*, 9524

**DOI:** 10.3390/ijms26199552

**Published:** 2025-09-30

**Authors:** Yaoning Chen, Wenjian Gong, Zhiyuan Zhang, Jianfeng Zhou, Deng-Guang Yu, Tao Yi

**Affiliations:** 1 School of Materials and Chemistry, University of Shanghai for Science and Technology, Shanghai 200093, China; 2 Faculty of Health Sciences and Sports, Macao Polytechnic University, Macau 999078, China

## Error in Figure 6 and Its Caption

In the original publication [1], there was a mistake in “Figure 6. XRD patterns of the three raw components (RES, VPV, and CA) and the different kinds of electrospun nanofibers (F1, F2, and F3)”, as published. The authors want to change the initial Figure 6 with a more clearer one to avoid possibly misleading the readers and correct the mistake of “VPV” in the caption to “PVP”. The corrected caption is “Figure 6. XRD patterns of the three raw components (RES, PVP, and CA) and the different kinds of electrospun nanofibers (F1, F2, and F3)”.

The XRD patterns of nanofibers F1, F2, and F3 and their noises are highly similar, which may mislead the readers. Thus, the preparations of samples and the XRD tests were repeated, and the new data were plotted overlapped in the graphic, the shape of the curves of F1, F2, and F3 being similar with the ones in [1], with a slightly more visible delimitation among them, the similarity being a common phenomenon for the amorphous polymer-based nanocomposites [2].

The authors state that the scientific conclusions are unaffected. This correction was approved by the Academic Editors. The original publication has also been updated, the original XRD data being available from the authors upon request.

**Figure 6:**
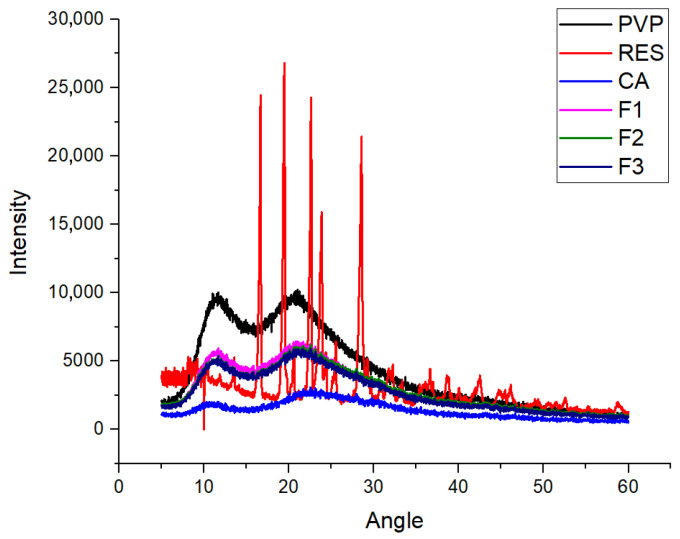
XRD patterns of the three raw components (RES, PVP, and CA) and the different kinds of electrospun nanofibers (F1, F2, and F3).

## References

[1] Chen Y., Gong W., Zhang Z., Zhou J., Yu D.-G., Yi T. (2024). Reverse Gradient Distributions of Drug and Polymer Molecules Within Electrospun Core–Shell Nanofibers for Sustained Release. Int. J. Mol. Sci..

[2] Sanchez-Vazquez B., Amaral A.J.R., Yu D.G., Pasparakis G., Williams G.R. (2025). Electrosprayed Janus Particles for Combined Photo-Chemotherapy. AAPS PharmSciTech.

